# Evaluation of 3D-printer settings for producing personal protective equipment

**DOI:** 10.2217/3dp-2021-0005

**Published:** 2021-08-20

**Authors:** Carson Studders, Ian Fraser, Joshua W Giles, Stephanie M Willerth

**Affiliations:** ^1^University of Victoria Department of Mechanical Engineering, Center for Biomedical Research, 3800 Finnerty Road, Victoria, BC V8W 2Y2, Canada

**Keywords:** 3D printing, additive manufacturing, COVID-19, face shield, material properties, personal protective equipment, polylactic acid, tensile testing

## Abstract

**Aim:** COVID-19 resulted in a shortage of personal protective equipment. Community members united to 3D-print face shield headbands to support local healthcare workers. This study examined factors altering print time and strength. **Materials & methods:** Combinations of infill density (50%, 100%), shell thickness (0.8, 1.2 mm), line width (0.2 mm, 0.4 mm), and layer height (0.1 mm, 0.2 mm) were evaluated through tensile testing, finite element analysis, and printing time. **Results:** Strength increased with increased infill (p < 0.001) and shell thickness (p < 0.001). Layer height had no effect on strength. Increasing line width increased strength (p < 0.001). **Discussion:** Increasing layer height and line width decreased print time by 50 and 39%, respectively. Increased shell thickness did not alter print time. These changes are recommended for printing.

## Background

COVID-19, caused by SARS-CoV-2, rapidly spread since its first case was recorded in December 2019 and resulted in a global pandemic [1]. Current research indicates that the virus is transmitted primarily through respiratory droplets [2]. The virus binds to angiotensin-converting enzyme 2 receptors located in the alveolar cells of the lungs after inhalation [1,3]. While symptoms vary between individuals, they most commonly manifest as respiratory distress, coughing, fever and fatigue [1,4,5]. Vascular issues, namely hypercoagulability increasing the risk of harmful blood clots, as well as neurological symptoms such as loss of taste and smell, have also been reported [6–10].

Many healthcare institutions around the world have been overwhelmed during the COVID-19 pandemic due to the high transmissibility, rate of fatality and surprise emergence of the disease. One effect of the surge of seriously ill patients now requiring professional medical attention is a shortage of personal protective equipment (PPE) for doctors, nurses and other healthcare professionals [11,12]. Globally, healthcare workers as foundational as primary care physicians have been unable to acquire sufficient PPE to protect themselves while they carry out their essential work [13–15]. Safety regulations regarding wearing PPE such as face shields, respirators, and medical masks to reduce the spread of COVID-19 through respiratory droplets are in place, but insufficient PPE supply can make these regulations difficult to follow, which compromises patient and provider safety [13,16]. Reusable physical barriers like plastic boxes, sheets, and hoods – designed to cover the patient and contain respiratory droplets during treatment – have been used in some instances, though research suggests they are not an appropriate substitute for conventional PPE [17].

Additive manufacturing of different types has been used in the medical field for years for patient-specific orthopedic implants, surgical guides and models for surgical training and visualization by producing objects from geometry encoded in a digital file, which can be derived from advanced medical imaging [18–20]. This manufacturing technique is widely lauded for generating complex parts without conventional restrictions, allowing personal customizability and facilitating rapid prototyping of otherwise expensive components [21]. In recent months, it has been used to create a variety of supplies within a short period of time to combat COVID-19-related shortages in mechanical ventilator components, face shield headbands and nasal swabs [22,23]. The large number of commercial and low-cost consumer products utilizing fused-deposition modeling (FDM) make it the most popular printing technology, thus it is likely to be the most effective for decentralized manufacturing efforts [24]. In FDM, a solid filament is melted and deposited in discrete layers by a heated nozzle onto a print bed, with parts being built up over a number of hours [25]. FDM printers have a large number of parameters that can be tuned, depending on the needs of the user: infill changes the proportion of solid to void space; shell thickness adjusts the width of the solid outer layer of the print; layer height determines how fine each layer of melted filament is; and line width adjusts the width of the melted polymer bead deposited by the nozzle. Many more parameters such as nozzle speed, infill pattern, and nozzle and bed temperature can also be adjusted. Generally, parameter changes that decrease print speed – such as increased infill – increase print quality. Parameters such as increased nozzle speed – which increase print speed – typically lead to decreased print quality.

Face shields printed using FDM can help curb the spread of the disease through respiratory droplets, with conventionally-made face shields shown to lower simulated large-droplet inhalation by up to 96% in comparison to unshielded, under specific circumstances [26]. The construction is very simple: the device itself consists of a headband, and a plastic shield that provides a physical barrier for the face. Face shields, specifically those printed from polylactic acid (PLA) polymer, have the additional advantage of being chemically sterilizable and reusable, which frees essential workers from further reliance on conventional supply chains for single-use versions of this article of PPE [27]. Members of the 3D-printing and healthcare communities have begun initiatives to print the headband components themselves for distribution in the community to circumvent the scarcity of conventionally manufactured supplies due to disruptions and shortages [28–30]. Such initiatives have been recognized at the institutional, local and national scale in countries like the UK [31–33].

At our institution, we partnered with the local health authority to form a COVID-19 response team of dedicated students, professionals, organizations and enthusiasts. We mobilized our set of 13 3D printers and acted as a hub for the distributed community network donating their own printed face shield headbands. Sanitized face-shield kits, including headbands, shields provided by a local manufacturer, and adjustable straps, were sent out to frontline workers in the community. The initiative was able to produce and supply over 5000 kits. Components printed in-house were made from PLA and printed on Ultimaker 2+ printers.

A balance between print strength and manufacturing speed must be found to enable community initiatives to produce face shields at a faster rate via 3D printing while still retaining good print quality and strength. Shell thickness, infill density, line width and layer height can all be tuned to manipulate print strength while theoretically impacting print time. The purpose of this study was to find the values for these parameters that represent a trade-off between the strength of the print and the time that it takes to print. The benchmark test will be how quickly a stack of four face-shield headbands can be printed using combinations of these parameters. We hypothesized that increasing shell thickness and infill density, and decreasing layer height, will lead to increases in print time and print strength, while increases in line width will decrease print time and increase strength.

## Materials & methods

### Sample preparation

Four permutations of infill density and shell thickness (Table 1) were tested independently from four permutations of line width and layer height (Table 2). This combination of permutations was selected to decrease the total number of samples that needed to be made to investigate the four parameters of interest. Each combination consisted of five samples, as in [34], to ensure a statistically relevant sample size; 40 samples total were printed. Values for the parameters were derived from those initially used by our initiative to print face shields.

**Table 1.** Infill density and shell thickness of each of the four groups tested for these parameters.

Group	Infill density (%)	Shell thickness (mm)
A	50	0.8
B	50	1.2
C	100	0.8
D	100	1.2

Each group consisted of five samples.

**Table 2.** Line width and layer height of each of the four groups tested with these parameters.

Group	Line width (mm)	Layer height (mm)
E	0.1	0.2
F	0.1	0.4
G	0.2	0.2
H	0.2	0.4

Each group consisted of five samples.

**Figure 1.**
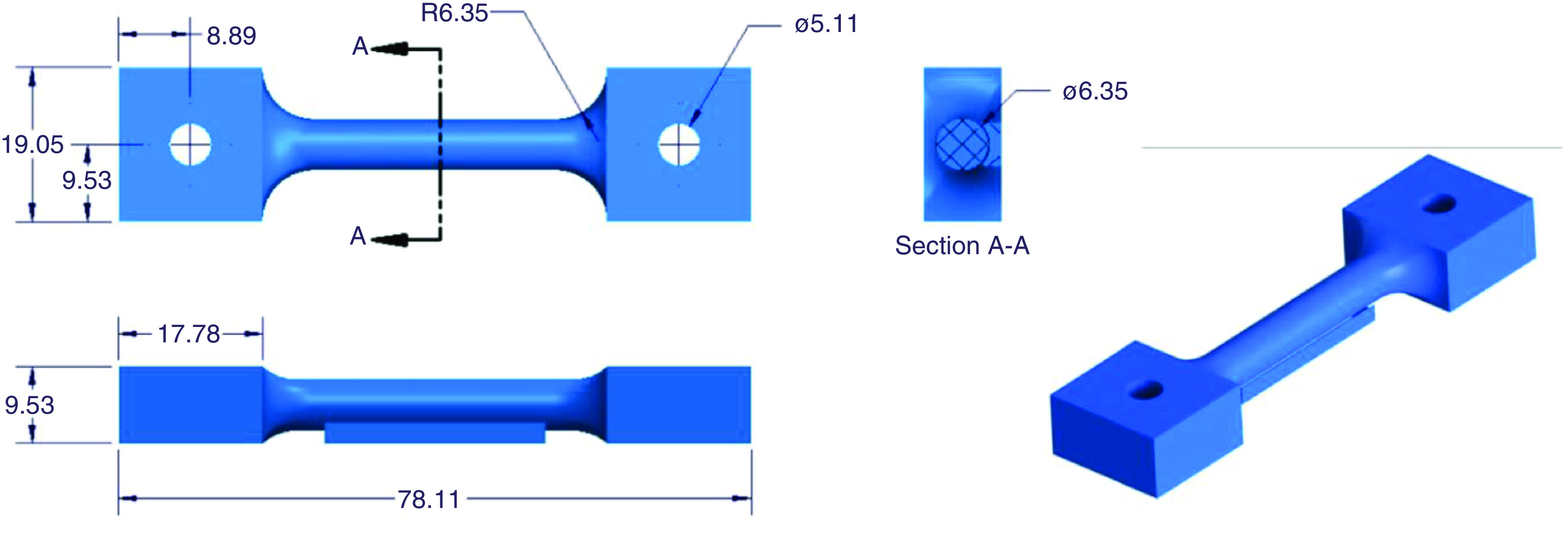
The 3D-printed polylactic acid sample used for tensile testing, with dimensions indicated in millimeters. The feature attached to the bottom in the bottom left view was used as a support during printing and removed before testing.

These print parameters were applied to test specimens with geometry as presented in Figure 1.

Test specimens were prepared as computer-aided design files in SolidWorks (Dassault Systèmes, Vélizy-Villacoublay, France), and exported as Standard Triangle Language (STL) files. These files were imported into CURA 4.6.1 for slicing and g-code generation (Ultimaker BV, Utrecht, Netherlands), and then printed using an Ultimaker 2+ 3D printer (Ultimaker BV) with 2.85 mm diameter Form Futura PLA blue filament (Form Futura, Nijmegen, Netherlands).

### Experimental testing

Each sample’s mass was measured using a Mettler B22 scale (Mettler Toledo, OH, USA) to ensure consistency between specimens in the same group. Specimen neck diameters were measured with Mastercraft digital calipers (Canadian Tire, ON, Canada) to account for variation within batches due to possible printer inconsistency, and to ensure accurate stress values were recorded. Test specimens were mounted in an MTI-10-K tensile testing machine (Measurements Technology Inc, LA, USA) and loaded at a constant velocity of 2.54 mm/s. Tensile testing was selected over bending due to the elastic nature of the material, equipment constraints, and because both tensile and bend tests result in normal stress in the tested component. Elongation was measured using an extensometer (model no. 3542-0100-010-ST, Epsilon Technology Corporation, WY, USA), with a gauge length of 1.0 in, and ±0.1 in (+10%) travel. Applied force, stress and elongation were recorded over time, and the highest stress value attained during the trial was used to compare groups.

### Statistical analysis of experimental data

Data were divided into two sets based on like parameters (groups A–D with varied infill and shell thickness in one, groups E–H with varied layer height and line width in the other). Levene’s test for homogeneity of variance was conducted prior to further analysis of the dependent variables of mass, diameter and stress. Both sets met the criteria for homogeneity of variance for each variable, except for the diameter of the layer height-line width classification (E–H). A one-way ANOVA and *post hoc* Tukey test were performed on those data that met the homogeneity of variance criteria. For the layer height-line width classification diameter, a Welch ANOVA was performed with a *post hoc* Games–Howell test. A two-way ANOVA was then performed on each set to determine the effect of each altered parameter on max stress at failure. An α value less than or equal to 0.05 was selected to denote statistical significance across all tests. Statistical analysis was done in SPSS (IBM Corp., NY, USA), and figures were made with GraphPad Prism (GraphPad Software Inc., CA, USA).

### Finite element analysis

An STL file of the freely-available, open-source Prusa headband was downloaded from the manufacturer’s website [35]. This particular model was selected as it was the first approved for use by the health authority we partnered with for this endeavor. The file was remeshed in 3-matic (Materialise, Leuven, Belgium), where adaptive remesh functionality was used to preserve the sharp edges and features present in the original geometry. Several meshes with different tetrahedral element edge length were generated (3, 2, 1.5 and 1 mm) for use in a mesh convergence analysis. Elements with a 1 mm edge length were ultimately used. Volumetric meshes were exported as .inp files and imported into Abaqus (Dassault Systèmes), an advanced finite element analysis software. Material properties in accordance with literature data were applied to the model as seen in Table 3.

**Table 3.** Material properties used to simulate polylactic acid in a simulation of the headband under load.

Property	Value	Ref.
Poisson’s ratio *v*	0.36	[36]
Young’s modulus *E* (MPa)	3.5 GPa	[36]

**Figure 2.**
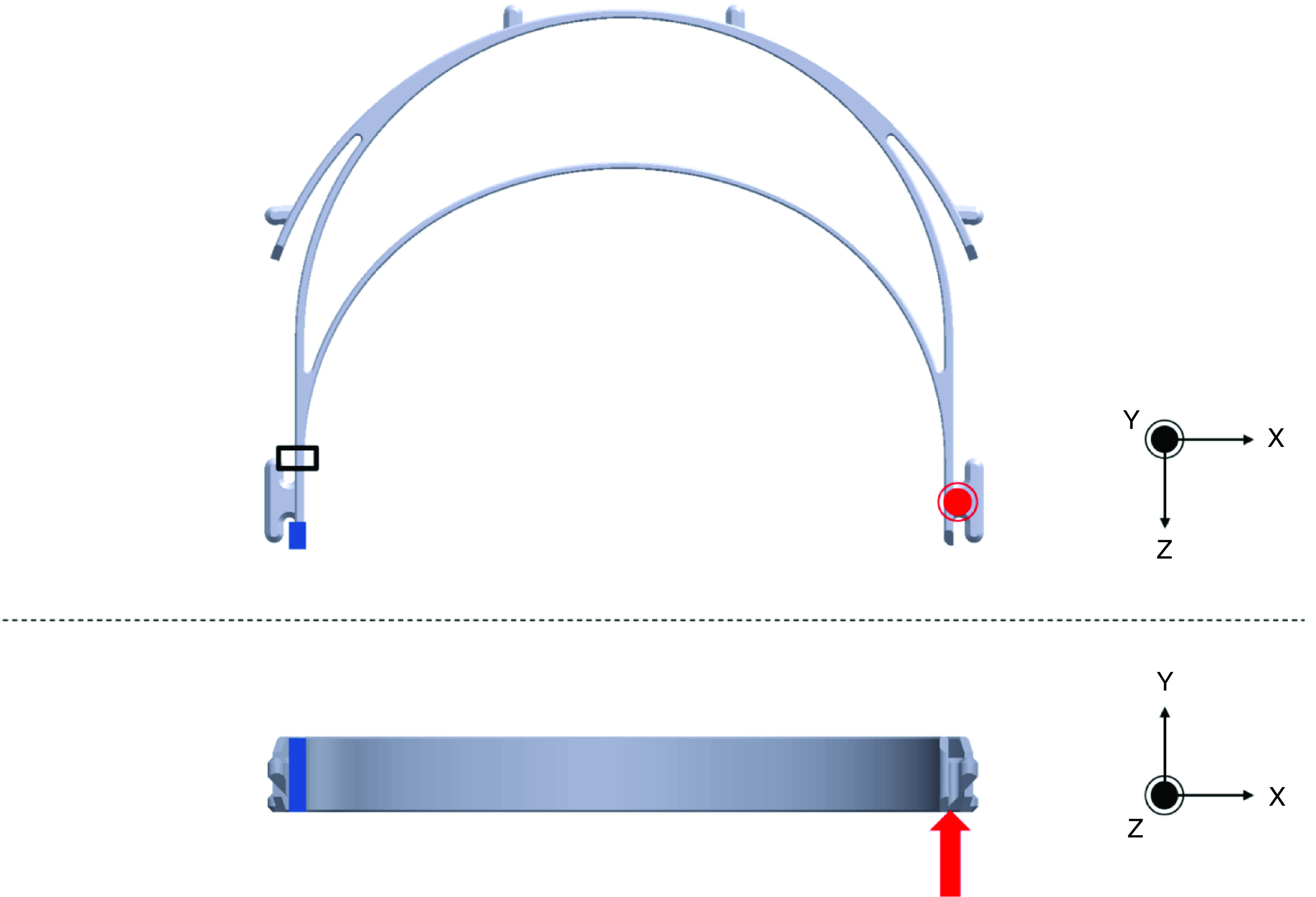
Load and boundary condition applied to the face-shield headband in finite element analysis simulation. Blue indicates the boundary condition (constrained from translation and rotation in all directions), red indicates a force (50N), and black denotes the critical area. The concentric circle symbol indicates an arrow coming out of the page.

The load and boundary conditions were selected to produce the highest-stress loading scenario, where torsion would be maximized. While this is not the only loading case the headband would be subjected to in reality, it is the one most likely to cause failure due to the excessive shear stress, which has a six-times greater multiplicative factor in the von Mises stress equation than normal stress [37]. The nodes on the back edge of the left side of the headband were constrained from rotation and translation in all directions while an upward shear load of 50N, force similar in magnitude to measured data of peak exertion index finger poking, pressing and pulling, was applied at the opposite end (Figure 2) [38]. While this process does not represent exactly how an individual may handle the headband, it provides an estimate for what could be considered excessively high forces during an object handing task such as putting on a face shield headband. Because of the force’s large magnitude, the computed safety factors will be lower than what would be the case during normal use. The critical region where failure was most likely was determined to be at the cross-section close to where the boundary condition was applied, though far enough away to minimize the influence of any cosmetic feature stress concentrations or singularities that would lead to unrealistically high stresses. To determine the headband’s safety factor, values of the von Mises stress from this location were analyzed and compared with the stress at failure measured experimentally during tensile tests. Nodal stress was exported for elements making up the cross-section, and the maximum value was determined and used for this comparison.

The safety factor was computed by comparing the maximum stress from this simulation to the stress at failure of each group using the equation

$$N=\frac{\sigma_{failure}}{\sigma^{\prime }}$$

### Printing time

Each set of parameters was applied to a stack of four PRUSA headbands sliced using CURA 4.6.1 to evaluate how they impacted print time. The same .STL file was used for this process as was used for FEA. These results were then compared with experimental testing and FEA results to determine which parameters were suitable for tuning.

## Results

### Experimental testing

Between groups A–D, statistically significant differences were detected in mass (p < 0.001), diameter (p = 0.001) and max stress (p < 0.001). Statistically significant differences in mass (p < 0.001), diameter (p < 0.001) and max stress (p = 0.004) were also detected in groups E–H. Results of the *post hoc* tests showing differences within sets (A–D or E–H) can be seen in Table 4 & Table 5.

**Table 4.** Results from measurement and tensile testing of samples with varying infill density and shell thickness.

		Mass (g)	Diameter (mm)	Max stress (MPa)
Group A	50% infill, 0.8 mm shell thickness	5.90 ± 0.10^BCD^	6.45 ± 0.02^D^	27.28 ± 0.54^BCD^
Group B	50% infill, 1.2 mm shell thickness	6.34 ± 0.08^ACD^	6.43 ± 0.05^D^	31.95 ± 1.28^ACD^
Group C	100% infill, 0.8 mm shell thickness	8.59 ± 0.09^AB^	6.41 ± 0.03^D^	44.58 ± 1.22^ABD^
Group D	100% infill, 1.2 mm shell thickness	8.71 ± 0.08^AB^	6.35 ± 0.02^ABC^	47.77 ± 1.39^ABC^

Values are average ± standard deviation. Statistically significant differences between the reported value and other values are denoted by the superscript letter of the statistically different group(s).

**Table 5.** Results from measurement and tensile testing of samples with varying layer height and line width.

		Mass (g)	Diameter (mm)	Max stress (MPa)
Group E	0.1 mm layer height, 0.2 mm line width	5.99 ± 0.15^GH^	6.35 ± 0.00^GH^	21.22 ± 4.16^H^
Group F	0.1 mm layer height, 0.4 mm line width	5.94 ± 0.18^G^	6.33 ± 0.04^H^	24.93 ± 1.62^G^
Group G	0.2 mm layer height, 0.2 mm line width	5.55 ± 0.05^EF^	6.38 ± 0.02^EH^	20.44 ± 1.46^FH^
Group H	0.2 mm layer height, 0.4 mm line width	5.72 ± 0.09^E^	6.46 ± 0.04^EFG^	25.96 ± 0.55^EG^

Values are average ± standard deviation. Statistically significant differences between the reported value and other values are denoted by the superscript letter of the statistically different group(s).

The results of Table 4 indicate the masses of samples A and B were different from each other and all others by a significant amount. Masses of groups C and D were significantly different from groups A and B, but not each other. Only the diameter of group D was significantly different from the others. Stress values for all groups were significantly different from one another. Masses in Table 5 have significant differences, though they do not follow as clear a pattern as those in Table 4. Group H’s diameter was significantly different from those of groups E and G, and the diameter of groups E and G were significantly different from each other. Stress values between groups E and F were not significantly different, while G and H were.

**Figure 3.**
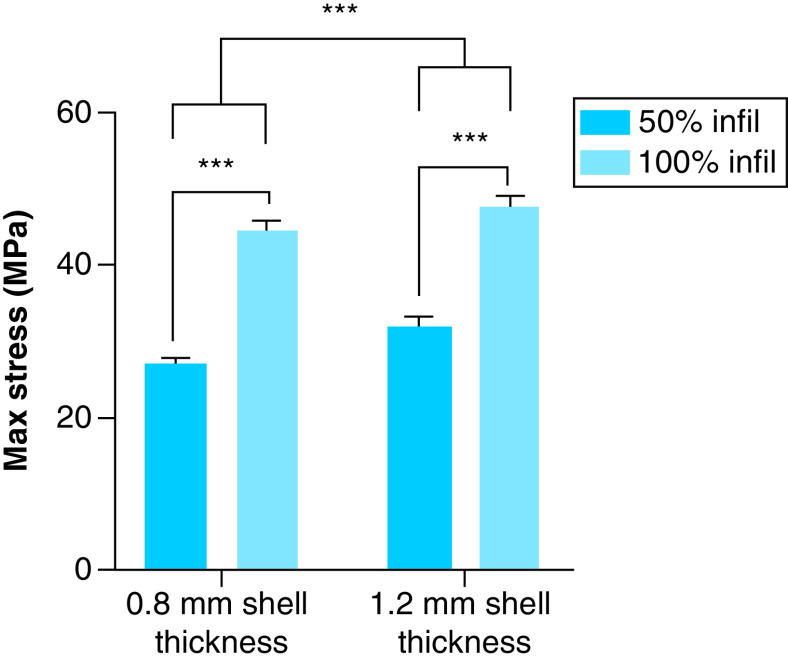
Results from tensile testing of samples with varying infill density and shell thickness. Statistical significance determined through a two-way ANOVA. Group means for samples with varying shell thickness were significantly different, as were those for samples with varying infill density. ***p < 0.001.

**Figure 4.**
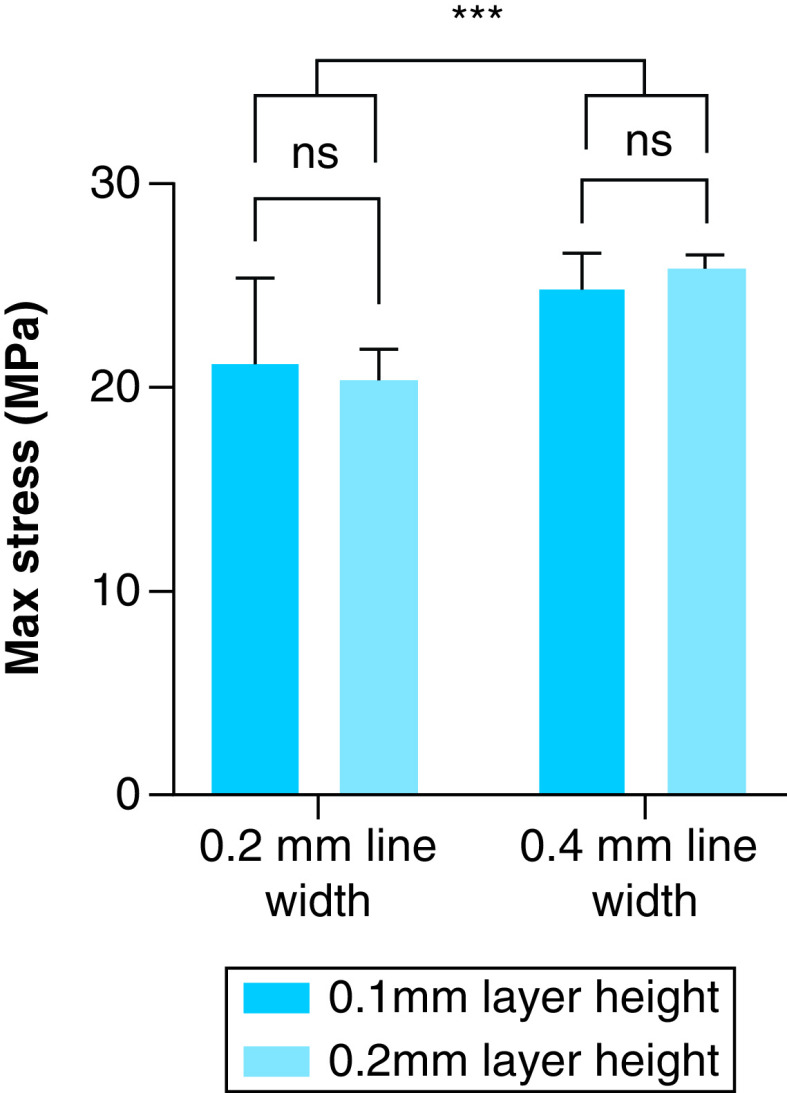
Results from testing 3D-printed samples with varying line width and layer height. Statistical significance determined through a two-way ANOVA. Group means of stress for samples with varying layer height were not significantly different, while groups with varying line width were. ***p < 0.001.

Further results from the two-way ANOVA investigating how altering each parameter affects material strength are presented in Figure 3 & Figure 4.

For groups with varying infill and shell thickness, significant differences between the means of all groups (p < 0.001), without significant interaction between them (p = 0.17), were detected. The mean stress at failure of groups with 100% infill was 46.18 MPa, while the mean stress at failure for groups with 50% infill was 29.62 MPa, for a total difference of 16.56 MPa. The mean stress at failure for groups with 0.8 mm shell thickness was 35.93 MPa, which differed from the mean failure stress of 39.86 MPa for groups with 1.2 mm shell thickness by 3.93 MPa.

For groups where line width and layer height were varied, a two-way ANOVA showed no statistical significance between means of max stress for the different layer heights (p = 0.91) and no interaction between layer height and line width (p = 0.41). Statistically significant differences were detected between different line width groups (p < 0.001), with mean values of 20.83 and 25.44 MPa for 0.2 mm line width and 0.4 mm line width, respectively.

### Finite element analysis

The maximum stress in nodes at the critical region cross-section was determined to be 84.12 MPa. Higher stress values occurred at cosmetic features of the headband not relevant to structural integrity, such as the branded lettering.

**Figure 5.**
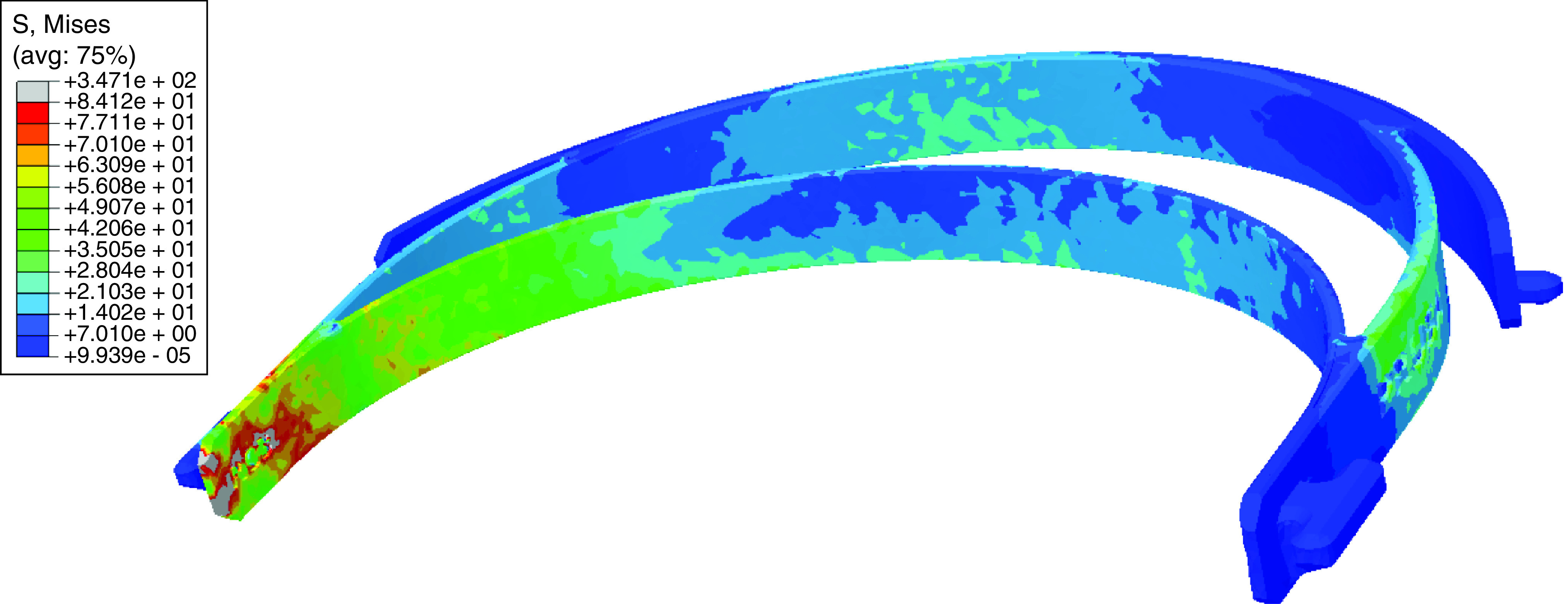
von Mises stress in the face shield headband under a 50N load with the left end fixed, as determined with finite element analysis. Stresses are in MPa. A threshold of 84.12 MPa, the max stress assessed in the critical area, was set to more accurately bound the heatmap. S: Stress.

Simulation results are shown in Figure 5.

Comparing the predicted maximum stress at the critical region to the strength at failure of each group yielded safety factors ranging from 0.24 to 0.57 (Table 6).

**Table 6.** Safety factors computed by comparing the strength at failure of groups A–D to the maximum stress in the critical area as determined using finite element analysis.

Group	Parameters	Average max stress (MPa)	Safety factor
A	50% infill, 0.8 mm shell thickness	27.28	0.32
B	50% infill, 1.2 mm shell thickness	31.95	0.38
C	100% infill, 0.8 mm shell thickness	44.58	0.53
D	100% infill, 1.2 mm shell thickness	47.77	0.57
E	0.1 mm layer height, 0.2 mm line width	21.22	0.25
F	0.1 mm layer height, 0.4 mm line width	24.93	0.30
G	0.2 mm layer height, 0.2 mm line width	20.44	0.24
H	0.2 mm layer height, 0.4 mm line width	25.96	0.31

### Printing time

Results from the investigation of print time can be seen in Table 7.

**Table 7.** Time to print a stack of four face-shield headbands using each of the groups of parameters as calculated by slicing in CURA 4.6.1.

Group	Parameters	Print time
A	50% infill, 0.8 mm shell thickness	16 h 35 m
B	50% infill, 1.2 mm shell thickness	16 h 35 m
C	100% infill, 0.8 mm shell thickness	20 h 29 m
D	100% infill, 1.2 mm shell thickness	20 h 29 m
E	0.1 mm layer height, 0.2 mm line width	49 h 28 m
F	0.1 mm layer height, 0.4 mm line width	29 h 42 m
G	0.2 mm layer height, 0.2 mm line width	24 h 29 m
H	0.2 mm layer height, 0.4 mm line width	14 h 57 m

## Discussion

Mass differences between groups A–D were more pronounced in samples with 50% infill density (A and B), as these groups were significantly different from all others. The masses of samples C and D, while significantly different from A and B, were not significantly different from each other. This may indicate that the additional mass added by increasing shell thickness was not within the window of standard deviation of mass purely added through infill. The results for groups A–D regarding max stress at failure follow an intuitive pattern, with all being significantly different from one another. The results for diameter, however, do not follow a clear pattern. The small standard deviations rule out measurement error, leaving printing anomalies as the likely source. The diameter of group D was significantly smaller, but this did not appear to compromise its strength as it was still significantly stronger than the other groups. Significant mass differences in groups E–G occurred primarily due to differing layer heights, as no significant differences were found between groups with the same line width. Group H had a significantly different diameter than E and G, and its larger cross-sectional area may have increased its strength, contributing to a significant difference between groups G and H, while E and F were not significantly different. The increased mean diameter of H, however, would only increase its max stress at failure by 3.5% compared with a sample with a nominal diameter; it is thus unlikely that this alone caused its higher strength.

Both infill density and shell thickness had significant effects on material strength, based on the results presented in Figure 3. As expected, higher infill density resulted in substantially greater strength, with 100% infill failing at stresses, on average, 16.56 MPa higher than 50% infill. While intuitive, this increase in tensile strength has also been reported in the literature [39]. The magnitude of the max stress for 100% infill with 0.8 and 1.2 mm shell thickness, 44.58 and 47.7 MPa, respectively, is similar to that reported in the literature for blue PLA printed with 100% infill using FDM, which had an average ultimate tensile strength of 54.11 MPa [40]. Differences can likely be attributed to other parameters those authors used during printing, which are not presented in the paper. Shell thickness also had a statistically significant impact on stress at failure, with means between groups varying by 3.93 MPa. This difference is likely due to the increased cross-sectional area with greater shell thickness, as there are fewer voids due to the selected infill pattern. Information specifically on the effect of shell thickness on part strength does not appear to be present in the literature.

Experimental testing of the groups with varying line width and layer height (Figure 4), revealed that layer height had little impact on max stress at failure. This similarity of performance agrees with existing research that showed our tensile samples that were printed flat, with varying layer heights had statistically significant but small (4.96%) differences in strength [34]. Interestingly, other research has determined that decreasing layer height can in fact increase strength [41]. These differences are likely due to a difference in values used for layer height: [41] used values between 0.1 to 0.6 mm, whereas [34] were between 0.06 to 0.24 mm – closer to the values and behavior to those in this present study. Alternate testing methodologies may also account for these differences: [41] performed a three-point bend test, while [34] performed both three-point bending and tensile tests. Increasing line width, on the other hand, led to an increase in stress at failure by an average of 4.61 MPa. This is likely a result of effectively increasing cross sectional area by decreasing the number of individual strands making up shelled areas of the print, which decreases overall void space. This mechanism has been previously described [41]. Researchers using varying nozzle diameters, and by extension varying line width, have demonstrated that an increased nozzle diameter/line width leads to increased tensile strength [41].

Combining the results from the FEA with those from experimental testing (Table 4 & Table 5) revealed that under a 50 N load producing torsional shear stress, all eight of the proposed combinations of parameters would fail. However, this does not mean that none of these parameters would produce a headband and thus face shield that provides an acceptable level of protection; the 50 N loading represents a much higher force than would typically be exerted on the headband, especially while in use, at the worst possible location. The results should instead help provide a ceiling for the force magnitude that can be applied without causing failure during handling. It may also be useful for those assembling face shields to know how much force can or should be applied during assembly, where forceful manipulation of the headband without causing damage is required to attach the plastic shield.

Combining the previous results with those from the test of time to print a stack of four headbands (Table 7) provides sufficient information to evaluate the overall quality of the different parameter combinations. Those groups with 0.1 mm layer height (groups E and F) had unacceptably long print times of 29 and 49 h for 0.2 and 0.4 mm line widths, respectively. Coupling this with the insignificant increases in strength that adjusting layer height provided, it is not recommended that smaller layer heights be used. Increasing line width from 0.2 to 0.4 mm not only reduced printing time, but also improved strength; thus, altering this parameter is recommended. In groups with varying shell thickness, it was observed that altering this parameter did not affect time to print as computed by the CURA 4.6.1 slicing algorithm, but did improve strength by a statistically significant amount. For these reasons, it is recommended that higher shell thickness be used so that print strength can be improved without sacrificing print time and thus manufacturing productivity. Finally, increasing infill density predictably increased print time, but led to significant improvements in strength and higher safety factors when subjected to excessive loading. This parameter can be adjusted with discretion, depending on the type of environment in which the user is operating. In situations where the headband could be forcibly knocked off or will be roughly handled, higher infill density is recommended. In environments where this is not likely to occur, it is likely that using lower infill density while making other strength improvements previously described would provide acceptable performance.

The present study is not without its weaknesses. Tensile testing colinear with the print strand axis, rather than bending, was done as this is the standard method for assessing failure strength and due to equipment limitations, though bending is a more likely loading condition for the headband. Given that both tests subject the component to tensile stress and ultimately provide a stress at failure that can be used in further analysis, this was deemed an appropriate substitution. In the FEA model, isotropic material behavior was assumed. This does not capture the full complexity of the anisotropic behavior of 3D-printed components, even with 100% infill, though it provided an appropriate estimate as validated through calculations with analytically derived formulas. The parameters tested were two discrete values on what is, in reality, an entire spectrum, which naturally limits the conclusions; the strength characteristics and print time between and beyond the values chosen, however, are expected to follow a similar pattern of behavior. Infill pattern was not investigated, though research into the effect of using different patterns at varying densities has been conducted, and demonstrated that performance differences exist between patterns, with some being stronger than others [39]. These differences could be evaluated in a later study to find additional ways to balance print time and strength.

## Conclusion

Results of the present study indicate that print strength and time can be controlled by adjusting infill density, shell thickness, line width and layer height. Using the values in this paper: increasing infill density led to increased printing time (24%), but significantly increased tensile strength; shell thickness increases did not change predicted printing time, but did significantly increase tensile strength; increasing line width led to decreased print time (-39%), and also increased tensile strength by a statistically significant amount; decreasing layer height had no significant effect on strength, but substantially increased print time (99%). Based on these findings, it is recommended that when printing face-shield headbands, line width and shell thickness be adjusted to improve strength and manufacturing output. Infill density should be increased at the discretion of the manufacturer or user to meet their specific environmental and usage needs. Decreasing layer height should not be used as a means to increase strength, especially if producing a high volume of headbands is the goal, though it can be increased as a means to increase production speed.

## Future perspective

The COVID-19 pandemic has pushed the healthcare community and beyond to find creative ways to navigate dire situations, including shortages of PPE. While the ideal solution is to structure supply chains and appropriately budget so that shortages do not occur in the future, it is also important to remember the strength of distributed community manufacturing and innovative open-source designs. Recent collaboration borne out of necessity will hopefully give rise to newfound levels of cooperation between healthcare professionals, engineers and members of the broader community that will help push toward innovative new technologies and strategies that can be made available to everyone.

The specific findings of this work, and the efforts of this work in general to characterize and optimize the quality of 3D-printed PPE, have broader applications beyond the COVID-19 pandemic. 3D printing is already being used in developing nations to provide low-cost prosthetic limbs. In locations that have limited access to PPE, 3D printers can be employed to produce it rather than relying on a conventional supply chain. While obtaining a 3D printer in these locations may be challenging, it would allow for a large supply of equipment once installed with no risk of shipping difficulties or delays, and has already been demonstrated to work in the case of prosthetics. Future research should focus on further optimizing print parameters so that PPE can be produced at low cost and made available where it is needed.

An opportunity for future work exists in several capacities. Other tunable 3D-printing parameters can be investigated to determine their effect on the strength characteristics of printed components with the goal of improving manufacturing capacity for PPE. Another possible avenue of exploration is formal mathematical optimization either using the parameters discussed in this work or with additional parameters, to determine which combination of values result in a print that truly optimizes the combination of strength and printing time.

**Summary points**

- Shortages of personal protective equipment have necessitated distributed community manufacturing of face-shield components via 3D printing.
- The impact of altering infill density, shell thickness, layer height and line width on print strength and time was investigated using a combination of tensile testing, finite element analysis and print time analysis.
- Increasing print infill led to a statistically significant increase in strength as measured by tensile testing: increasing infill from 50 to 100% increased print time by 24%, though it also increased time to print. Increasing print shell thickness significantly increased strength without changing measured printing time, and should thus be used to improve strength.
- Increasing line width from 0.2 to 0.4 mm decreased print time 39% and increased strength by a statistically significant amount, and should thus be altered for both strength and speed improvements.
- Decreasing layer height from 0.2 to 0.1 mm did not significantly change strength but increased printing time by 99%.
- When combinations of parameters were applied to a face shield headband finite element model and subject to a 50 N load, no samples had safety factors greater than one, though this does not necessarily indicate that these parameter combinations will not perform adequately under normal conditions.
- This research indicates that there are a variety of ways to increase the strength of printed face shield components and other 3D-printed parts without sacrificing time to print. We recommend increasing line width, layer height, and shell thickness, as they either decrease time to print, increase strength, or both.
